# Titanium Nitride Modified Fiber Optic Interferometer for Refractive Index Sensitivity Enhancement

**DOI:** 10.3390/s23115280

**Published:** 2023-06-02

**Authors:** Duo Yi, Bin Zhang, Youfu Geng, Xuejin Li

**Affiliations:** 1 College of Physics and Optoelectronic Engineering, Shenzhen University, Shenzhen 518060, China; 2 Institute of Translational Medicine, The First Affiliated Hospital (Shenzhen Second People’s Hospital), Shenzhen University, Health Science Center, Shenzhen 518060, China; 3 School of Science, Chinese University of Hong Kong, Shenzhen 518172, China

**Keywords:** titanium nitride, fiber optic interferometer, refractive index enhancement

## Abstract

As one of the most well-established biocompatible transition metal nitrides, titanium nitride (TiN) has been widely applied for fiber waveguide coupling device applications. This study proposes a TiN-modified fiber optic interferometer. Benefiting from the unique properties of TiN, including ultrathin nanolayer, high refractive index, and broad-spectrum optical absorption, the refractive index (RI) response of the interferometer is greatly enhanced, which is desired all the time in the field of biosensing. The experimental results show that the deposited TiN nanoparticles (NPs) can enhance the evanescent field excitation and modulate the effective RI difference of the interferometer, which eventually results in the RI response enhancement. Besides, after incorporating the TiN with different concentrations, the resonant wavelength and the RI responses of the interferometer are enhanced to varying degrees. Benefitting from this advantage, the sensing performances, including sensitivity and measurement range, can be flexibly adapted based on different detection requirements. Since RI response can effectively reflect the detection ability of biosensors, the proposed TiN-sensitized fiber optic interferometer can be potentially applied for high-sensitive biosensing applications.

## 1. Introduction

In the past years, nanomaterials have attracted great interest due to their excellent optical, electronic, and mechanical properties, and they bring breakthroughs to almost all related fields, including optoelectronics, chemistry, and biology [1,2]. Among them, transition metal nitrides have been widely reported due to their high electrical conductivity, outstanding chemical stability, and excellent mechanical strength [3,4,5]. As one of the most well-established biocompatible transition metal nitrides, TiN shows high stability and hardness and excellent biocompatibility, and it has been discovered as a plasmonic material with outstanding plasmonic performances [6,7]. More importantly, TiN exhibits unique optical characteristics such as broad-spectrum optical absorption, high-efficiency photothermal conversion, and large nonlinear optical response at communication bands, etc. [8,9], and the introduction of TiN has brought new vitality to fiber optic device applications, including ultrafast photonics [10,11], high-speed optical transmission [12], and temperature measurement [13].

Although tremendous efforts have been dedicated towards developing high-performance TiN-integrated fiber optic devices, challenges still remain to achieve high-performance sensing devices, particularly for biosensing applications. As is well known, various biological actions (DNA binding, antigen−antibody binding, etc.) are usually accompanied by RI variation, and RI response sensitivity is an important parameter that is widely used to quantitatively characterize the detection ability of biosensors. However, the RI variation during biological reaction processes is usually tiny, and the traditional fiber optic biosensor is still difficult to meet the ultra-low detection limit biosensing application (e.g., lower than nMol or even pMol). Thus, fiber optic biosensors with an enhanced RI sensitivity response are strongly desired all the time. Among them, fiber optic interferometer and fiber optic surface plasmon resonance sensor are the two main candidates. As for the fiber optic interferometer, the ambient RI can effectively modulate the interference phase, and therefore we can realize the ambient RI detection by locating the resonant wavelength of the interference spectrum. However, the RI sensitivity of the traditional fiber optic interferometer is relatively low, and RI response enhancement is essential. The mainstream methods for RI response enhancement are based on the modification of the fiber structure itself, such as fiber tapering [14], side-polishing [15], and cladding etching [16]. However, the fiber structure becomes fragile after modification, which limits the actual applications. In addition, the RI response of the fiber sensor can also be enhanced by incorporating novel functional materials. In recent years, emerging 2D materials such as graphene [17] and MXenes [18] have been reported, and they can effectively realize RI response enhancement of the fiber optic biosensors owing to their abundance of surface functional groups. However, until now, the TiN-based fiber optic RI sensing devices have been rarely reported, to the best of our knowledge, and the corresponding RI response enhancement mechanism is still unknown. Overcoming these doubts leads to the objectives of this study.

In this study, we propose a TiN-incorporated fiber optic interferometer, and its RI response sensitivity is enhanced owing to the unique properties of TiN, including ultrathin nanolayer, high refractive index, and broad-spectrum optical absorption, etc. Firstly, the transmission spectra of the interferometer before/after TiN deposition are compared to analyze the effect of TiN deposition on the modulation of optical properties of the interferometer. The main parameters of the spectrum, including free spectrum range (FSR) and fringe contrast ratio, etc., are discussed, respectively. Next, the effect of different TiN concentrations on the RI response enhancement of the fiber device is discussed. The experimental results show that the resonant wavelength of the interferometer dip can be effectively modulated by incorporating TiN, which eventually results in the RI response enhancement. Besides, the experimental results also demonstrate that the enhanced RI response can be dynamically adjusted by incorporating TiN with different concentrations, signifying that the sensing performances (sensitivity, measurement range) of the TiN-incorporated fiber optic interferometer can be flexibly adapted according to the different detection requirements, showing a unique advantage when compared with the traditional fiber optic biosensors. We believe that the presented study can promote the development of TiN-based biosensing technology, particularly for low-concentration detection applications.

## 2. Materials and Methods

### 2.1. TiN Characterizations

**Figure 1:**
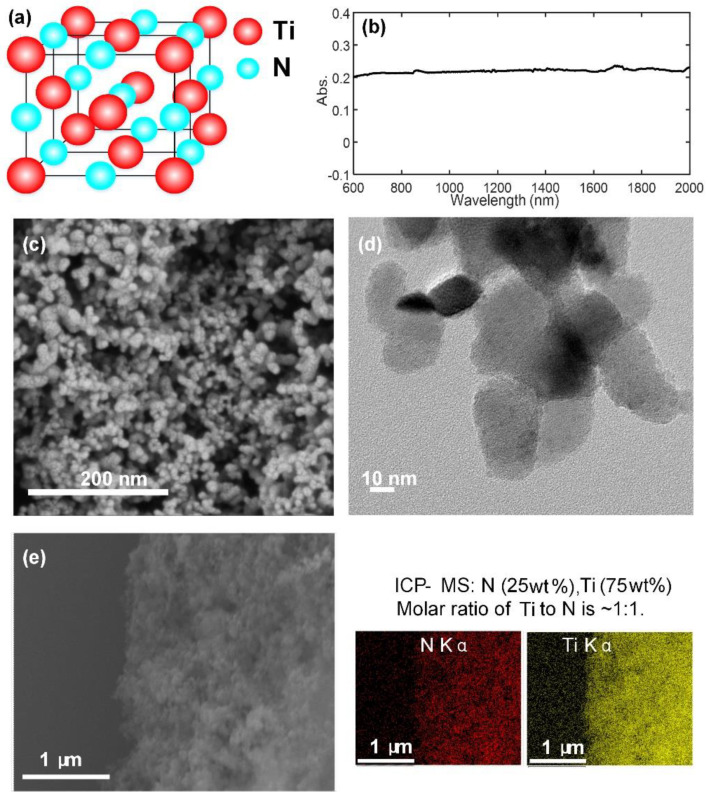
Material characterizations of the TiN sample. (**a**) Face−centered cubic structure. (**b**) Optical absorption behavior. (**c**) SEM image. (**d**) TEM image. (**e**) EDX mapping of the as−synthesized TiN NPs.

In this work, uniformly dispersed TiN NPs were fabricated via a hydrothermal process, followed by a high-temperature treatment in ammonia gas flow. The specific TiN preparation can be found in Supporting Information Section S1. After dispersing the as-synthesized TiN NPs into isopropanol, a series of characterizations were conducted, as indicated in Figure 1. Figure 1a represents the cubic structure of TiN, and Figure 1b shows the optical absorption behavior of TiN solution. Since the optical absorption behavior becomes more significant as concentration increases, herein, a relatively low concentration, i.e., 20 μg/mL is selected to make the result believable. It demonstrates that the TiN NPs own excellent linear optical absorption within a broad-spectrum range of 600–2000 nm. In terms of morphology, monodispersed TiN NPs are successfully synthesized with a size of around 20 nm, as can be observed in the SEM (scanning electron microscopy) (Figure 1c) and TEM (transmission electron microscope) (Figure 1d) images. To check the composition of the as-synthesized NPs, EDX (energy-dispersive X-ray spectroscopy) and ICP-MS (inductively coupled plasma-mass spectrometry) were further performed, and the results are listed in Figure 1e. Ti and N elements are well-distributed across the material skeleton, and the 1:1 molar ratio proves that the chemical composition is TiN.

### 2.2. Fabrication of Fiber Optic Interferometer

**Figure 2:**
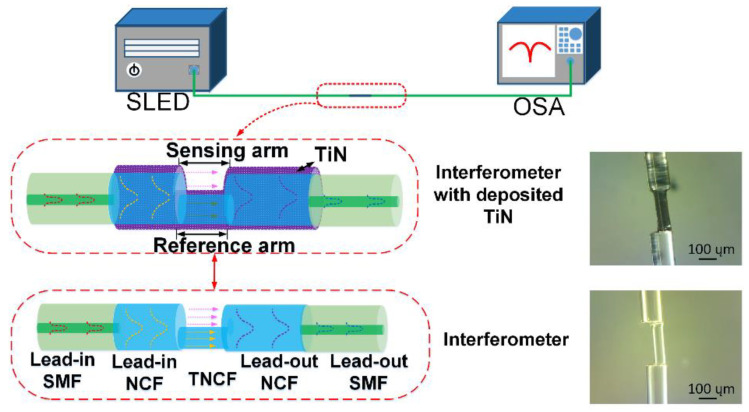
Diagram of fiber optic interferometer and experimental setup.

In this study, a fiber optic interferometer was fabricated, and the interferometer structure is shown in Figure 2. The sensor consists of lead-in Single-Mode-Fiber (SMF, 9/125 µm)—lead-in No-Core-Fiber (NCF, 125 µm)—Thin-No-Core-Fiber (TNCF, 62.5 µm)—lead-out No-Core-Fiber (NCF, 125 µm)—lead-out Single-Mode-Fiber (SMF, 9/125 µm). Among them, both TNCF and NCF are made of pure silica with air as cladding instead of traditional core/cladding multimode structure, the special waveguide structure allows for the sufficient excitation of evanescent wave around the fiber surface, and the related introductions of NCF can be found in our previous works [19]. The TNCF (length: 233 μm) is spliced between lead-in NCF and lead-out NCF with a lateral offset of 31.2 µm, and the fusion splicing parameters are set as follows: arc power ~180 bits, arc duration ~200 ms. In addition, the lead-in NCF and lead-out NCF (length: 1 mm) are spliced with lead-in SMF and lead-out SMF, respectively, and the fusion splicing parameters are set as follows: arc power ~ 308 bits, arc duration ~800 ms. They operate as the light beam expander/focus, respectively, to improve the waveguide coupling efficiency between the two connected fibers. Otherwise, the interference performance will obviously deteriorate when the lateral-offset distance slightly deviates from the optimal value, which further increases the fabrication complexity of the device [20]. Finally, it should be explained that the TNCF is cleaved under microscope during the fabrication process, and its splicing length is accurately controlled to the micron level. The TNCF length is designed as only several hundred microns to ensure that the light power in both the sensing arm and reference arm are approximately equal, which ensures a high fringe contrast.

The proposed fiber optic sensor operates as a Mach-Zehnder interferometer. When the light transmits through the interface between the lead-in NCF and TNCF, part of the light continues to transmit along the TNCF, forming the reference arm; the rest of the light transmits through the air cavity around TNCF, forming the measurement arm. Two parts of light recouple into the lead-out SMF, and interference occurs. Besides, when the interferometer is used for the aqueous solution test, the air cavity will be replaced by the liquid solution. Under this condition, the solution fills the air cavity, which operates as both light transmission channel and sensing channel. The dip wavelength of the proposed interferometer can be expressed as:

(1) λ=2ΔnL/(2m+1)

where *Δn* is the effective RI difference between the measurement and reference arms, *L* is the length of TNCF, and *m* is the interference order. Besides, *FSR* describes the wavelength gap between two adjacent interference dips. *FSR* has a close relationship with the effective RI difference *Δn*, and it is defined as:

(2) $$FSR=\lambda^{2}/\Delta nL$$

The proposed fiber optic interferometer exhibits two main advantages. Firstly, the dimensions of the sensing arm/reference arm are at the micrometer level, and the compact structure facilitates future integration applications. Secondly, the RI difference of the interferometer is directly determined by the TNCF and the tested aqueous solution, and a tiny RI change of the tested solution can significantly modulate the effective RI difference of the interferometer, which eventually results in a high RI response.

### 2.3. Fabrication of the TiN-Incorporated Fiber Optic Interferometer

In this study, the exfoliated TiN NPs were dispersed in isopropyl alcohol, and they were transferred onto the fiber surface via the optical deposition method. Additionally, Figure 2 shows the comparison of surface morphology of the fiber optic sensor by optical microscope. The bare fiber optic shows a smooth surface, while the obvious non-transparent deposit can be clearly observed after TiN incorporation. In the following analyses, to evaluate the effect of TiN deposition on the transmission spectrum of the fiber optic interferometer, the TiN sample with concentrations of 2.50 mg/mL was prepared. Besides, to evaluate the effect of different TiN concentrations on the RI response enhancement of the interferometer, the TiN samples with different concentrations of 2.50 mg/mL, 1.66 mg/mL, and 1.25 mg/mL were prepared. They were dropped onto the three identical interferometers, respectively, which were finally converted into TiN overlayers after volatilization.

### 2.4. Measurement System

To evaluate the effect of TiN deposition on the RI response enhancement of the fiber optic interferometer, an experimental setup was built up, as indicated in Figure 2. During the test, the two ends of the TiN-incorporated interferometer were connected with the broad-bandwidth SLED light source (Fiberlake Co., Ltd., Shenzhen, China, WBB400008SFA) and the optical spectrum analyzer (OSA, Yokogawa Co., Ltd., Tokyo, Japan, AQ6370D). When the system works, the OSA resolution is set as 0.1 nm, and the incident light launched from the SLED transmits into the interferometer. The excited evanescent wave interacts with the deposited TiN, and it generates a strong light–TiN interaction. As a result, the optical properties of the fiber are modulated, which modifies the transmission spectrum of the interferometric light. Finally, the transmission spectra were experimentally acquired for the discussions.

## 3. Results

### 3.1. Optical Property Modulation of the Fiber Optic Interferometer by Adding the TiN Layer

**Figure 3:**
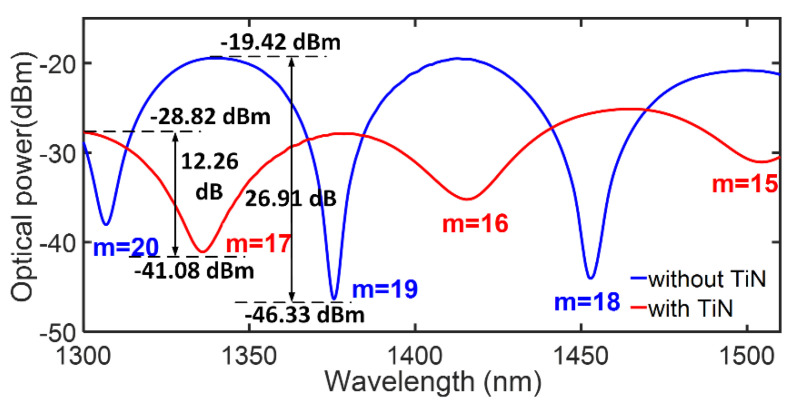
Comparison of transmission spectra of the interferometer before/after TiN deposition.

Firstly, the interferometer was immersed in pure water (RI value: 1.3347), and the transmission spectra of the interferometer before/after TiN deposition were experimentally acquired for comparison, as indicated in Figure 3. For the conventional fiber optic interferometer without TiN deposition, two *FSR*s were recorded as 68.7 nm and 77.4 nm, respectively, and the average *FSR* in the whole spectrum range was determined to be 73.05 nm. After TiN NPs were deposited, the corresponding *FSR*s were recorded as 79.6 nm and 88.8 nm, and the average *FSR* was determined to be 84.2 nm. According to Equation (2), the variation of *FSR* signifies that the effective RI difference of the interferometer has been modulated due to the TiN deposition. Assuming *L* = 233 µm, *λ* = 1400 nm, the effective RI difference *Δn* can be calculated. For the bare interferometer without TiN deposition, *Δn* was estimated as 0.1152, while this value decreases to 0.0999 after TiN is deposited, i.e., the effective RI modulation of the interferometer is determined to be 0.0153 due to the TiN deposition. We should explain that both effective RI modulation and *FSR* variation can be used to evaluate the transfer effect and actual deposition amount of TiN. A larger variation of RI modulation or *FSR* signifies that more TiN NPs are successfully deposited onto the fiber surface, which has a greater impact on the interferometer’s performances. In addition, by locating the specific resonant wavelengths of the interferometer dips, we can calculate the corresponding interference orders, and the results are also indicated in Figure 3. Obviously, the whole spectrum shows a significant blue-shift of dip wavelength due to TiN deposition.

Following this, the mechanism of TiN−induced effective RI modulation was briefly discussed. The process mainly involves the interaction between TNCF, TiN, and analyte solution. After TiN NPs were deposited, the TiN and the analyte solution can be considered as a hybrid sensing layer, and the introduction of TiN modulates the overall effective RI of the hybrid sensing layer. Specifically speaking, the effective RI of the hybrid sensing layer can be expressed as *n_eff_ = f_analyte_ × n_analyte_ + f_TiN_ × n_TiN_*, where *f_analyte_* and *f_TiN_* are the volume fraction of analyte solution and TiN, respectively, and *n_analyte_*, *n_TiN_* are their corresponding effective RIs. Obviously, when TiN with high RI is deposited, the volume fraction of the TiN increases correspondingly, and it leads to a high effective RI of the hybrid sensing layer. On the other hand, the effective mode RI of TNCF is considered to be a constant. As a result, the effective RI difference of interferometric light *Δn* decreases and *FSR* becomes larger, which confirms the results in the previous paragraph.

Next, the other parameters, including insertion loss, fringe contrast ratio, and figure of merit, are discussed. Firstly, the insertion loss becomes higher after TiN NPs are deposited, and it increases from 19.42 dB to 28.82 dB. This result can be explained by the deposited TiN NPs absorbing an extra part of the evanescent wave energy, which increases the transmission loss. Secondly, the fringe contrast ratio is also modified after TiN deposition, and it decreases from 26.91 dB to 12.26 dB. This result can also be explained by the light absorption caused by the deposited TiN NPs, which adjusts the optical power ratio between the sensing arm and reference arm of the interferometer, resulting in the modulation of the fringe contrast ratio. Finally, the figure of merit (FOM) is determined by the full width at half maxima (FWHM) of the spectrum. In Figure 3, the FOMs show varying degrees of deterioration due to the FSR enlargement. Despite that, the spectrum after TiN deposition still exhibits enough high resolution for resonant wavelength location.

### 3.2. RI Response Enhancement of the Fiber Optic Interferometer by Adding the TiN Layer

**Figure 4:**
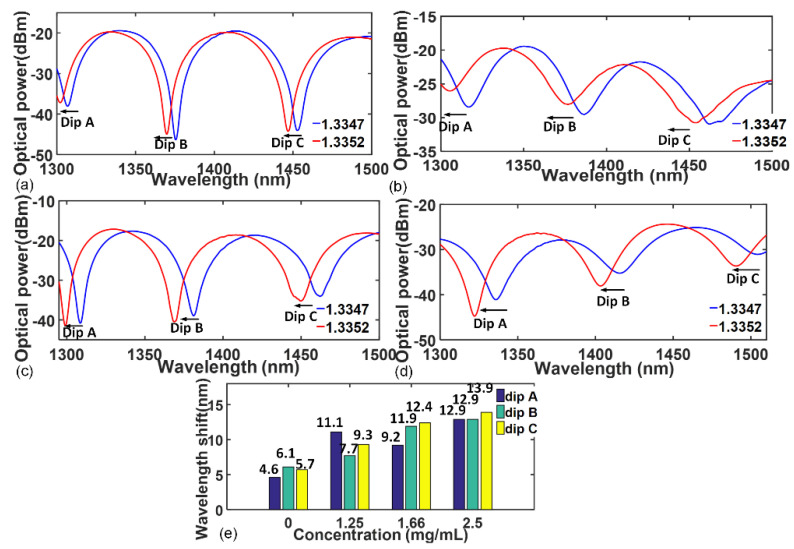
RI−induced wavelength shifts for the interferometer with different TiN concentrations. (**a**) 0 mg/mL. (**b**) 1.25 mg/mL. (**c**) 1.66 mg/mL. (**d**) 2.5 mg/mL. (**e**) Wavelength shift summary of different interference dips.

In the following part, we evaluate the effect of different TiN concentrations on the RI response of the interferometer. Herein, two different RI solutions (1.3347 and 1.3352) were prepared to evaluate the RI-induced wavelength shifts of the interferometers. Figure 4a–e show the experimentally acquired wavelength shift results. From Figure 4a,b, we can see that the wavelength shift becomes larger after TiN is deposited, which signifies an enhanced RI response. Theoretically speaking, the RI response enhancement is mainly impacted by two factors. Firstly, the ultra-thin, ultra-smooth TiN layer with nanometer size provides space for light penetration, and the surface evanescent wave can penetrate both TiN layer and analyte solution. As a result, the TiN layer provides additional light absorption, which is beneficial to enhance the evanescent wave–TiN interaction and eventually improves the excitation efficiency of the surface evanescent wave. In addition, a previous study reported that the RI sensitivity of the fiber optic interferometer is proportional to the change in propagation constants and the evanescent field [17]. Therefore, the RI response can be enhanced owing to the larger excitation of surface evanescent wave. Secondly, the deposited TiN layer can also effectively modulate the RI distribution between the two contacted mediums (TNCF and RI solution). The RI-induced wavelength shift can be calculated via *S = Δλ/Δn_analyte_*, where *Δn_analyte_* represents the RI increment of the analyte solution, i.e., 1.3352 − 1.3347 = 0.0005, and it is a constant. However, *Δλ* in this study represents the wavelength shifts caused by the hybrid sensing layers instead of the single sensing layer. As discussed previously, the introduction of TiN with high RI can effectively increase the overall effective RI of the hybrid sensing layer, which eventually leads to a larger wavelength shift when compared with that caused by the single sensing layer. Thus, an enhanced RI-induced wavelength shift S can be obtained owing to the TiN deposition.

In addition, from Figure 4b–d, we can also observe a clear trend—that a larger TiN concentration leads to a more significant wavelength shift. The specific wavelength shift values of different interference dips are summarized in Figure 4e. The average wavelength shifts were determined to be 5.5 nm, 9.4 nm, 11.2 nm, and 13.2 nm for different TiN concentrations of 0, 1.25, 1.66, and 2.5 mg/mL, and several conclusions can be summarized. Firstly, the wavelength shifts of the three interference dips are enhanced, which confirms that deposited TiN NPs exhibit good optical absorption properties within a wide near-infrared spectrum range. Secondly, a larger TiN concentration means a larger probability of effective deposition of TiN onto the fiber surface. As a result, the volume fraction of TiN NPs increases, which leads to a rise in overall effective RI of the hybrid sensing layer and a smaller effective RI difference *Δn* of the interferometer. Finally, according to Equation (1), a more significant RI-induced wavelength shift can be observed.

However, we should also note that the average wavelength shifts in Figure 4e are not strictly proportional to the TiN concentration. Besides, the distribution of wavelength shifts between different interference dips shows a degree of randomness. This can be mainly explained by the deposition method and optical absorption property of the TiN. In this study, the optical deposition method is used, and it is quite difficult to accurately control the deposition uniformity even though the solution concentration is fixed. Meanwhile, the optical absorption coefficient of the deposited TiN NPs is not a constant in a broad spectral range of communication band, and it slightly varies from different wavelength regions. The above two reasons lead to the randomness of wavelength shifts between different interference dips. Furthermore, although Figure 4e demonstrates an enhanced RI response with an increasing TiN concentration, the increments of average wavelength shifts tend to be smaller, which signifies that the RI response enhancement effect will not continuously improve by increasing TiN concentration. This phenomenon can be mainly explained by the evanescent field of the interferometer. As discussed in the last paragraph, when TiN starts to deposit onto the fiber surface, the ultrathin nanolayer can improve the evanescent wave excitation, which eventually leads to the RI response enhancement. However, when the TiN nanolayer becomes thicker, it gradually exceeds the effective penetration depth of the evanescent wave. As a result, the evanescent field excitation tends to be stable, and the RI response enhancement effect becomes less significant. It is difficult to precisely determine the specific value of effective penetration depth due to the lack of reported data for the optical constant of TiN with nanometer size, and the general value is believed to be around several tens to hundreds of nanometers.

**Figure 5:**
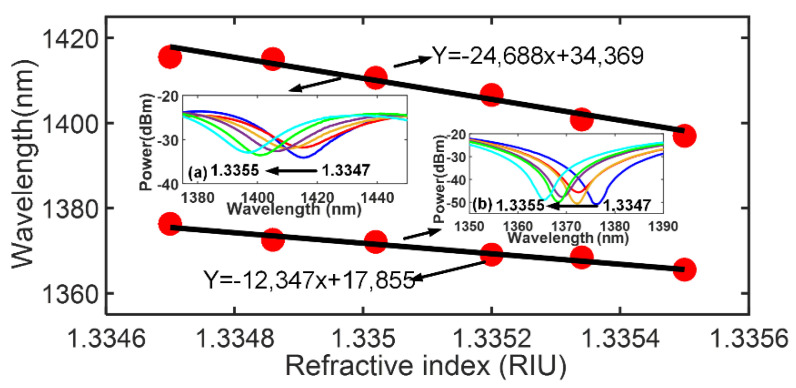
RI response sensitivity enhancement of the TiN−incorporated interferometer (a) transmission spectra of the TiN−incorporated interferometer (b) transmission spectra of the bare interferometer.

In the following part, the TiN-incorporated interferometer with a concentration of 2.5 mg/mL was selected, and dip B Figure 4 was taken as an example to evaluate the specific RI response sensitivity. The different RI solutions (1.3347~1.3355 with an increment of approximately 0.00016) were prepared for the RI sensitivity test, and the results are shown in Figure 5. The increase in the ambient RI leads to the decrease in the effective RI difference of the interferometer. As a result, it shows the blue-shift of resonant wavelength according to Equation (1). The RI response sensitivity of the bare interferometer is 12,347 nm/RIU, while it improves to 24,688 nm/RIU for the TiN-incorporated interferometer, which is approximately twice that of the bare interferometer. This result confirms that the RI response sensitivity can be effectively improved by incorporating TiN nanolayer. The enhanced RI sensitivity significantly improved over the previously reported fiber optic RI interferometers/nanomaterial-coated fiber optic RI sensors, and Table 1 shows the performance comparison.

Finally, the application scope of the proposed TiN-incorporated fiber sensor is briefly discussed. From the results in Figure 3, Figure 4 and Figure 5, we found that the deposited TiN NPs can flexibly modulate the resonant wavelength of interference dip and FSR. Benefitting from this advantage, the proposed fiber device can be potentially applied for tunable optical filtering. Besides, for the conventional fiber optic interferometer, its RI measurement range is determined by the ratio between FSR and dip RI sensitivity. Once the device dimension is determined, its performance parameters, including RI sensitivity and measurement range, are all fixed. This study proposes an alternative solution to overcome this limit, i.e., by incorporating TiN with different concentrations, the RI response sensitivity can be enhanced to varying degrees. In future studies, by specializing in TNCF length and the incorporated TiN concentration, we can fabricate a standardized sensing device, while the device performances such as sensitivity or measurement range can be flexibly adjusted according to different application requirements. This can largely extend the application scope of the sensing device. However, the TiN modification also leads to varying degrees of deterioration of spectrum parameters such as insertion loss, fringe contrast ratio, and FOM, and therefore optimized structure design and material deposition methods should be further evaluated. In addition, the long-term stability of the sensor is important to meet good sensing requirements, and a metrological study is necessary. These will lead to further investigations in the future.

## 4. Conclusions

In summary, taking advantage of the unique properties of TiN, including ultrathin nanolayer, high refractive index, and broad-spectrum optical absorption, a RI sensitivity enhanced-fiber optic interferometer is proposed. The experimental results demonstrate that the resonant wavelength of the fiber optic interferometer is effectively modulated as TiN deposits. This result confirms the effective RI modulation of the fiber interferometer due to the introduction of TiN. Benefitting from this advantage, the proposed interferometer can potentially be used for tunable optical filtering applications. Besides, the deposited TiN NPs were verified to realize the RI response enhancement owing to the effective RI modulation and stronger evanescent field excitation. With the increasing deposited TiN concentration, the RI responses of the TiN-based interferometer show varying degrees of enhancement, signifying that the performance parameters such as sensitivity and measurement range of the sensing device can be flexibly adjusted according to the actual requirements without modifying the sensor structure. This work is expected to greatly benefit the researchers in the field of high-performance fiber optic biosensors based on functional nanomaterials.

## Figures and Tables

**Table 1:** Comparison of the sensing performances with the state-of-art fiber optic RI interferometers/nanomaterial-coated fiber optic RI sensors.

Sensor Type/Year	Sensitivity (nm/RIU)	Range (RIU)	Reference
Side-polished MZ interferometer/2021	213.479	1.33269–1.39716	[21]
Air gap-based MZ interferometer/2022	226.8	1.43–1.45	[22]
FP interferometer + Al_2_O_3_ nanofilm/2015	6008	1.3371–1.3474	[23]
NCF + Ag + graphene/2018	3936.8	1.3330–1.3737	[24]
SPR + MXene/2020	2180.2	1.3343–1.3658	[18]
Presented work	24,688	1.3347–1.3355	

## Data Availability

Data are contained within the article or Supplementary Material.

## Supplementary Materials

The following supporting information can be downloaded at: https://www.mdpi.com/article/10.3390/s23115280/s1. Section S1. Preparation of TiN materials.
