# Supplementary material for: Titanium Nitride Modified Fiber Optic Interferometer for Refractive Index Sensitivity Enhancement
Source: Sensors (Basel). 2023 Jun 2;23(11):5280. doi: 10.3390/s23115280 (PMC10256003; doi:10.3390/s23115280)
Supplement: Supplementary file 1 [file sensors-23-05280-s001.zip › sensors-2408895-supplementary.pdf]

Supporting Information

Titanium nitride modified fiber optic interferometer for refractive index sensitivity enhancement

Duo yi, Bin Zhang, Youfu Geng, Xuejin Li

Section S1. Preparation of TiN materials

Tetrabutyl titanate, glycerol, methanol, ethanol, sodium borohydride, sodium hydroxide and ethyl ether were purchased from Macklin co. ltd. Then, the preparation of pristine TiN was achieved following a solvothermal procedure. In brief, 500 mg of tetrabutyl titanate, 12 mL of glycerol, 20 mL of methanol and 12 mL of ethyl ether were mixed under vigorous stirring for 1 hour, followed by a solvothermal process at 150℃ for 15 hours. The resultant products were collected via centrifugation at 12000 rpm for 15 hours and washed with ethanol for 3 times, and then were dried in a vacuum oven at 70℃ overnight. Next, the product was placed in a programmed controlled heating tubular furnace, and the precursors was annealed in ammonia gas flow at 450℃ for 2 hours, and then annealed at 600℃ for another 2 hours with a controlled heating process (25℃ to 450℃, 5℃/min; 450-700℃, 3℃/min).
